# Molecular and structural dissection of *Psalmopoeus pulcher* venom reveals cysteine-rich peptides that target acid-sensing ion channel 1a

**DOI:** 10.1016/j.jbc.2026.113347

**Published:** 2026-07-21

**Authors:** Zhaotun Hu, Jiatian Quan, Jiamin Yu, Dongfang Tang, Peng Feng, Bingxin Xiao, Yuhong Guo, Yu Zeng, Hang Zhu, Yichong Ning, Zhonghua Liu, Piao Zhao, Zhen Xiao

**Affiliations:** 1 The National and Local Joint Engineering Laboratory of Animal Peptide Drug Development, College of Life Sciences, and Peptide and Small Molecule Drug R&D Platform, Furong Laboratory, Hunan Normal University, Changsha, China; 2 Key Laboratory of Research and Utilization of Ethnomedicinal Plant Resources of Hunan Province, College of Biological and Food Engineering, Huaihua University, Huaihua, China; 3 College of Chemistry and Bioengineering, Hunan University of Science and Engineering, Yongzhou, China; 4 Yuelushan Laboratory, Changsha, China; 5 Institute of Plant Protection, Hunan Academy of Agricultural Sciences, Changsha, China

**Keywords:** *Psalmopoeus pulcher*, cDNA library, peptide toxin, ASIC1a, structure prediction

## Abstract

Spider venoms are rich sources of peptide toxins that modulate ion channels and receptors. Although individual toxins from the genus *Psalmopoeus* have been shown to be critical for probing the function of certain ion channels, the overall venom composition of this genus remains poorly characterized. In this study, we investigated the molecular and structural features of peptide toxins from the spider *Psalmopoeus pulcher*, focusing on peptide diversity and structural architecture. The molecular diversity of *P. pulcher* venom was examined through mass spectrometric analysis combined with construction of a venom-gland cDNA library. A total of 495 high-quality expressed sequence tags were obtained, including 357 toxin-like expressed sequence tags, encoding 69 nonredundant toxin precursors containing signal peptides. Based on sequence homology and cysteine frameworks, these precursors were classified into 11 families. Structural predictions using AlphaFold 3 revealed marked diversity in cysteine connectivity and folding patterns among the predicted mature peptides, encompassing canonical inhibitory cystine knot scaffolds, disulfide-directed hairpin motifs, peptides with expanded disulfide networks, and a subset of precursors predicted to adopt complex, multidomain architectures. Functional exploration combining sequence analysis, molecular docking, and limited experimental validation identified Pp1a, a psalmotoxin-1-like peptide encoded by multiple precursors, as an inhibitor of acid-sensing ion channel 1a. Collectively, this study expands current knowledge of the molecular diversity and structural characteristics of *P. pulcher* venom peptide and provides a foundation for further functional exploration and utilization of this venom-derived peptide resource.

Spider venoms represent one of the richest natural reservoirs of bioactive peptides, shaped through hundreds of millions of years of evolution as highly specialized tools that allow spiders to incapacitate prey, deter predators, and adapt to diverse ecological niches (1, 2). These peptides, typically cysteine-rich and stabilized by multiple disulfide bonds, possess exceptional structural stability and diverse three-dimensional motifs that underpin these ecological functions. Beyond their ecological roles, venom-derived peptides have emerged as valuable molecular probes and promising leads for ion channel structure-function studies, investigations of cellular mechanisms, and the development of insecticidal or therapeutic agents (3, 4). Although the World Spider Catalog currently lists more than 50,000 described spider species, most remain known only at the taxonomic level, with few species having any biochemical or molecular characterization of their venom peptides (1). This knowledge gap arises from several challenges, including enormous species diversity, limited venom yields, low abundance of bioactive components, and difficulties in specimen collection. Moreover, habitat fragmentation and environmental pressures threaten many spider populations, underscoring the urgency of documenting their venom diversity before it is irreversibly lost. The construction of venom-gland cDNA libraries has proven particularly useful for cataloging toxin-like precursors, especially when venom quantities are limited, providing an alternative approach to transcriptomic studies (5). Such libraries have been established for multiple spider genera, expanding the known molecular diversity of spider toxins. Coupled with molecular docking, phage display, yeast two-hybrid systems, cDNA-level repertoires can guide the prediction of bioactivity prior to labor-intensive experimental validation (6, 7, 8). Importantly, naturally evolved venom peptides continue to offer structural and functional features that remain difficult to fully recapitulate by current computational design strategies.

The genus *Psalmopoeus* (family Theraphosidae) comprises a small group of tarantulas, with approximately 20 recognized species. Research on *Psalmopoeus cambridgei* has been relatively well-developed, particularly regarding its modulation of acid-sensing ion channel 1a (ASIC1a) and transient receptor potential vanilloid 1 (TRPV1) channels. Crude venom from this species inhibits ASIC1a, highlighting the potential of ASIC1a inhibition as a neuroprotective strategy in ischemic stroke, and activates TRPV1 channels, which may mediate pain signaling (9, 10). Mechanistic studies indicate that these effects are primarily attributable to two well-characterized classes of toxins: Psalmotoxin-1 (PcTx1), a selective ASIC1a inhibitor, and Vanillotoxins (VaTx1/2/3), which activate TRPV1 (9, 11). Both toxins were fully sequenced using Edman degradation. These molecules have been employed as molecular tool to gain mechanistic insights into the physiological and pathophysiological roles of the ASIC1a and TRPV1 channels (12, 13, 14, 15). In particular, PcTx1 has become one of the most extensively studied spider peptide toxins and serves as a benchmark molecule for investigating ASIC1a structure–function relationships and channel pharmacology. To our knowledge, despite these advances, the overall venom composition of *P. cambridgei* remains incompletely characterized, and very little is known about other species within the genus *Psalmopoeus*. Recent transcriptomic analysis of *Psalmopoeus reduncus* identified hundreds of putative peptide sequences (16); however, many appear incomplete, and their potential functions remain largely unexplored. These knowledge gaps underscore the need for systematic characterization of venom peptides in understudied *Psalmopoeus* species.

In this study, we aimed to investigate the molecular and structural features of peptide toxins from the venom of *Psalmopoeus pulcher*, a species within the genus that remains poorly characterized. To this end, a venom-gland cDNA library was constructed to survey toxin-like precursors, and reverse-phase high-performance liquid chromatography (RP-HPLC) coupled with mass spectrometry was employed to characterize the peptide composition of the crude venom. Sequence homology analysis and molecular docking were further applied to provide preliminary functional insights into selected toxin families and to guide the selection of candidates for experimental evaluation. Through this integrated strategy, we aimed to establish a molecular framework for understanding the peptide diversity of *P. pulcher* venom and to provide a basis for subsequent functional and mechanistic studies of spider-derived peptide toxins.

## Results and discussion

### Molecular diversity of venom peptides in *P. pulcher*

**Figure 1:**
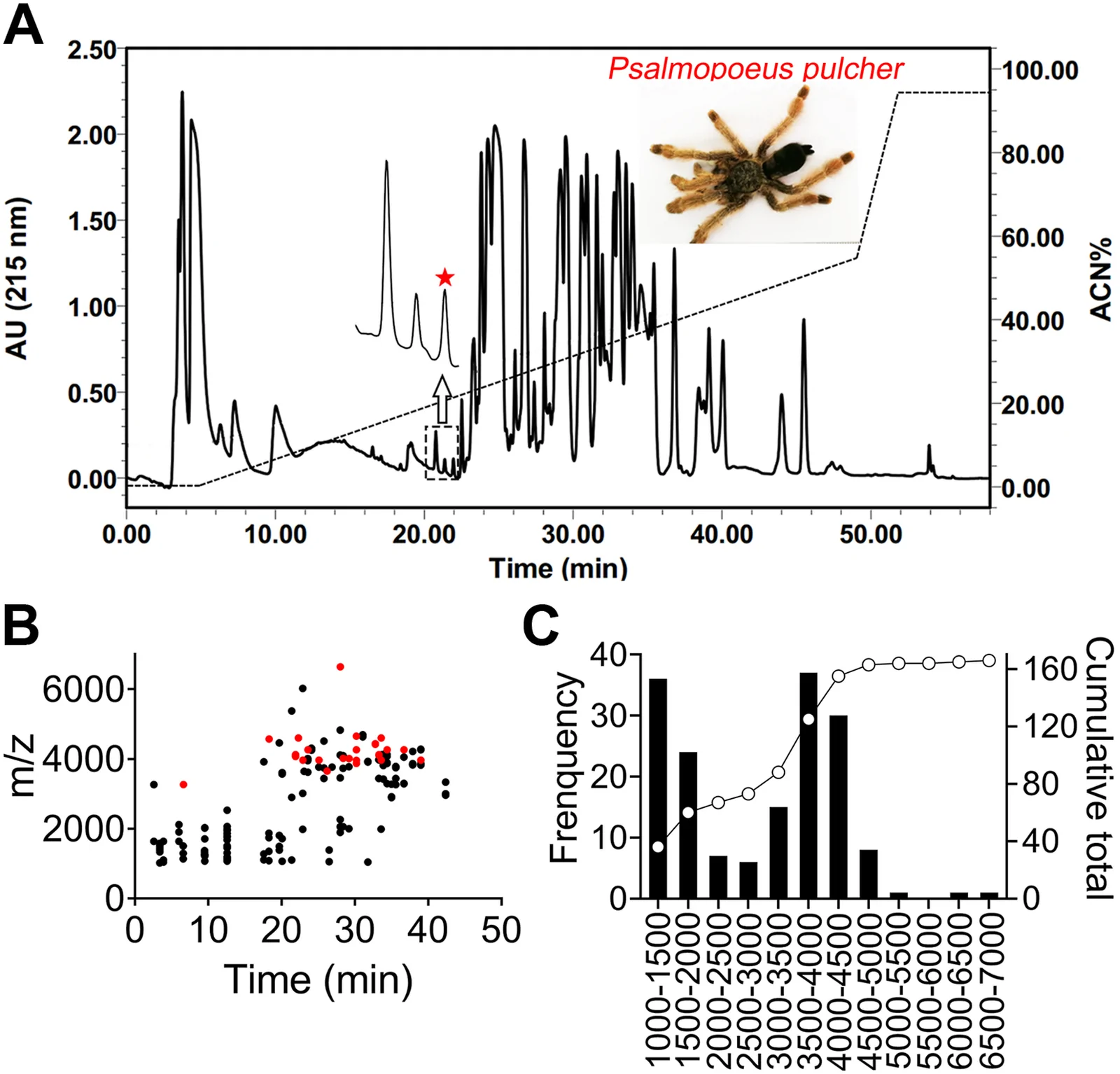
**Peptides diversity of *P. pulcher* venom.***A*, RP-HPLC profile of *P. pulcher* venom, the asterisk indicated the peak containing Pp1a. The inset shows the appearance of *P. pulcher* spider. *B*, molecular mass distribution of individual RP-HPLC fractions from *P. pulcher* venom determined by MALDI-TOF MS. *Red data points* represent MS peaks that are potentially assigned to putative mature peptides based on molecular mass matching. *C*, histograms represent the abundance of peptide toxins in the venom, grouped into 500 Da molecular mass classes, with overlaid curve indicating cumulative total of spider venom components. MALDI-TOF MS, matrix-assisted laser desorption/ionization time-of-flight mass spectrometry; RP-HPLC, reverse-phase high-performance liquid chromatography.

*P. pulcher* (commonly referred to as the Panama blonde tarantula) is a member of the genus *Psalmopoeus* (17) (Fig. 1*A*, inset). To characterize the molecular diversity of venom peptide components in *P. pulcher* venom, crude venom was subjected to RP-HPLC, which resolved the venom into multiple distinct chromatographic components (Fig. 1*A*). Each component was subsequently analyzed by matrix-assisted laser desorption/ionization time-of-flight mass spectrometry (MALDI-TOF MS) to determine its peptide mass composition. In total, 166 unique masses were detected, spanning approximately 1 to 7 kDa, reflecting a broad spectrum of venom peptides (Fig. 1*B*). The majority of detected components (∼98%) fell within the 1 to 5 kDa range, whereas peptides with molecular masses exceeding 5 kDa accounted for only ∼2% of the total signals. It should be noted, however, that the limited ionization efficiency and detection sensitivity of MALDI-TOF MS for higher-molecular-weight peptides may lead to an underrepresentation of larger venom peptides, implying that additional high-mass components remain to be discovered (Fig. 1*C*). In addition to peptides, spider venom is known to contain small biogenic amines and various enzymatic proteins; however, these classes of molecules were not further analyzed here, as they fall beyond the focus of this study. Taken together, the mass-spectrometry results underscore the substantial molecular complexity of *P. pulcher* venom, reflecting the diverse suite of bioactive compounds present at the proteomic level.

### Construction of the *P. pulcher* venom-gland cDNA library and cluster analysis

**Figure 2:**
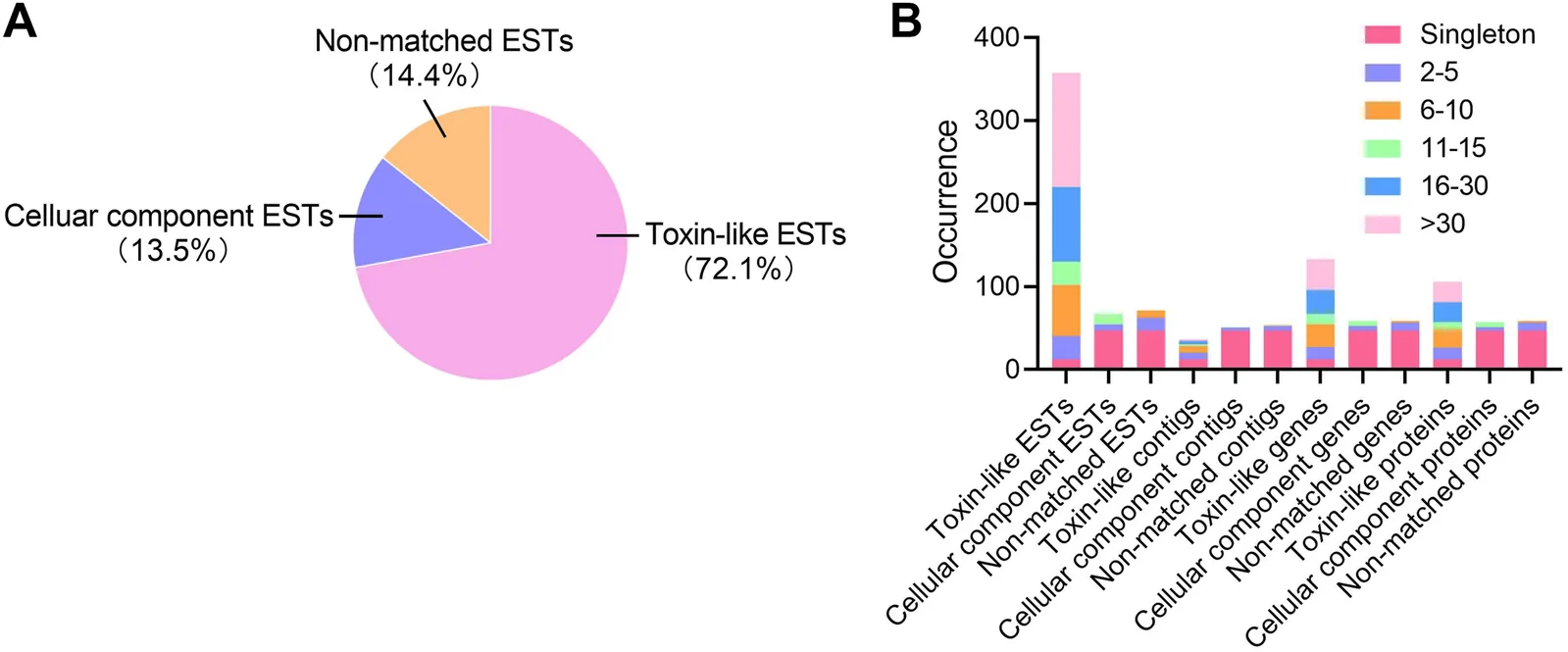
**Classification and cluster analysis of the cDNA library of *P. pulcher* spider venom gland.***A*, relative proportions of three transcript categories, toxin-like ESTs, cellular component ESTs, and nonmatched ESTs. *B*, occurrence of ESTs, contigs, unique genes, and proteins from the three functionally annotated categories among different cluster groups. EST, expressed sequence tag.

Sequencing of the *P. pulcher* venom-gland cDNA library yielded 495 high-quality expressed sequence tags (ESTs). These sequences were classified into three major categories based on BLAST searches against the NR protein database. As shown in Figure 2*A*, toxin-like ESTs were strongly enriched, comprising 357 sequences (72.1% of the total). ESTs associated with cellular components accounted for 67 sequences (13.5%), whereas the remaining 71 ESTs (14.4%) showed no significant similarity to known entries and were therefore designated as unclassified.

Firstly, 357 toxin-like ESTs are grouped into 36 clusters, as shown in Figure 2*B* including 12 singletons and 24 contigs: (1) eight contigs of size 2 to 5 contain 28 ESTs, which represent 15 unique genes encoding 14 proteins; (2) eight contigs of size 6 to 10 contain 62 ESTs, which represent 27 unique genes encoding 21 proteins; (3) two contigs of size 11 to 15 contain 28 ESTs, which represent 13 unique genes encoding nine proteins; (4) four contigs of size 16 to 30 contain 90 ESTs, which represent 29 unique genes encoding 24 proteins; (5) two contigs of size > 30 contain 137 ESTs, which represent 37 unique genes encoding 23 proteins.

The cellular component ESTs are grouped into 51 clusters, including 47 singletons and four contigs: (1) three contigs of size 2 to 5 contain 7 ESTs, which represent five unique genes encoding four proteins; (2) one contig of size 11 to 15 contains 13 ESTs, which represent six unique genes encoding six proteins.

The nonmatched ESTs are grouped into 53 clusters, including 47 singletons and six contigs: (1) five contigs of size 2 to 5 contain 15 ESTs, which represent nine unique genes encoding nine proteins; (2) one contig of size 6 to 10 contains 9 ESTs, which represent two unique genes encoding two proteins.

Among the 357 toxin-like ESTs identified from the venom-gland cDNA library, redundancy filtering yielded 103 unique peptide-precursor transcripts. Of these, 69 sequences encoded canonical secreted precursors carrying an N-terminal signal peptide, whereas 34 additional toxin-like precursors lacked a detectable signal peptide. Given that cysteine-rich venom peptides are typically secreted *via* the classical secretory pathway into the venom gland lumen, our analyses here focused on the signal peptide–containing precursors (18, 19). Nevertheless, we do not rule out the possibility that the precursors lacking a signal peptide may also possess important biological functions. Overall, the successfully constructed cDNA library revealed that toxin precursor-encoding transcripts constitute a major component of gene expression in the venom gland of *P. pulcher*.

### Comparative analysis and classification of toxin precursors from *P. pulcher*

A total of 69 NR precursors with signal peptide were retained for downstream analysis. A preliminary comparative analysis was further performed with toxin precursor reported in *P. reduncus* (16), a congeneric species of *P. pulcher*. The *P. reduncus* dataset contained a larger number of toxin precursor sequences, likely due to its greater sequencing depth (Fig. S1*A*). To ensure comparability, only *P. reduncus* peptides starting with Met at the N terminus were considered to ensure accurate comparison. In both species, most toxin precursor peptides ranged from 81 to 90 amino acids in length, indicating a broadly similar size distribution (Fig. S1*B*). However, pairwise sequence comparison revealed limited conservation between the two species, with only 1.3% of precursor peptide pairs sharing >50% sequence identity (Fig. S1*C*). Representative PcTx1-and VaTx1-like precursor from *Psalmopoeus*, including *P. pulcher*, *P. cambridgei*, and *P. reduncus*, were subsequently subjected to detailed comparative analyses.

**Figure 3:**
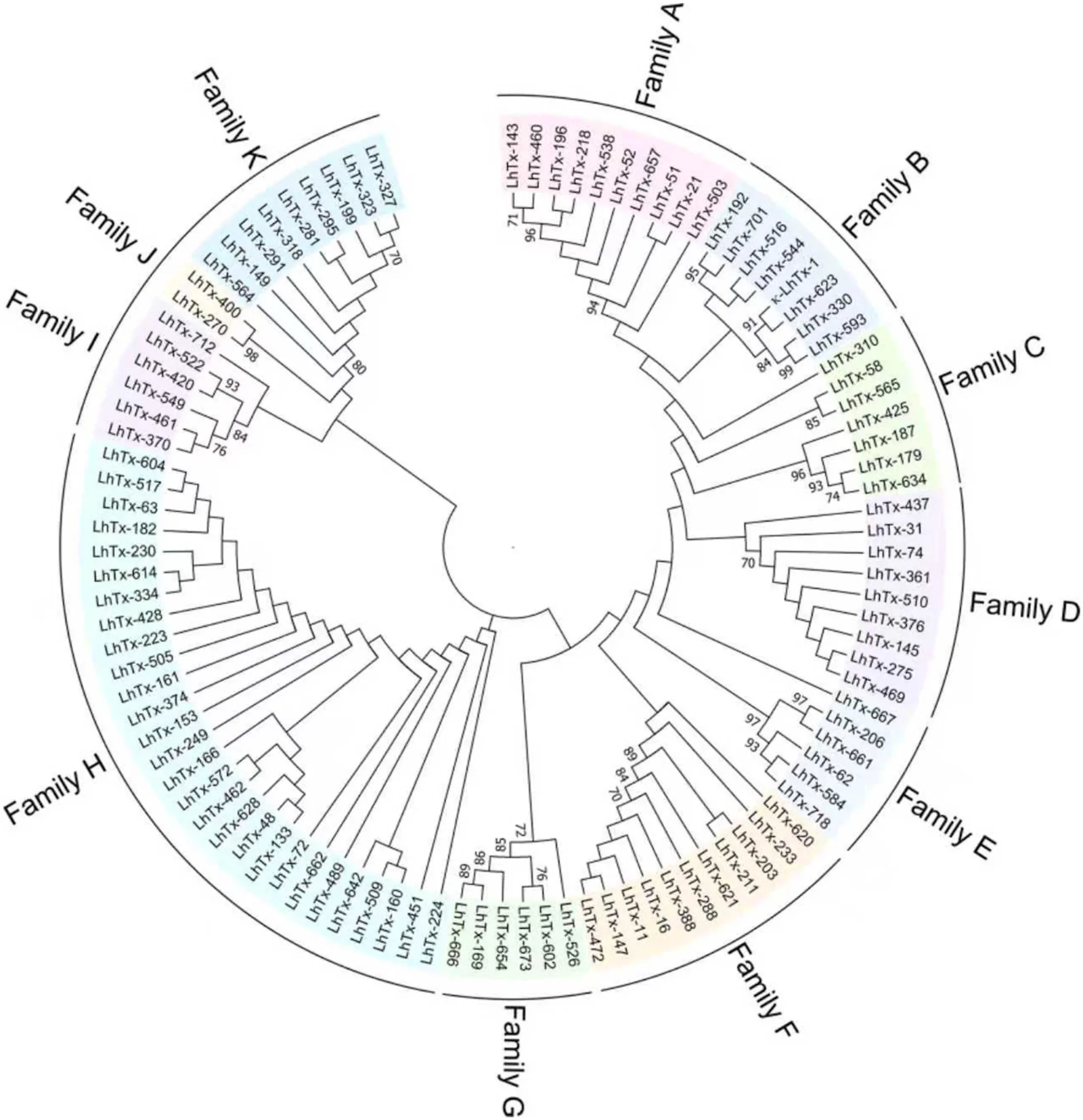
**Phylogenetic analysis of putative toxin precursors identified from the *P. pulcher* venom-gland cDNA library.** A total of 69 toxin precursors were clustered into 11 families based on sequence similarity and cysteine frameworks. The phylogenetic relationships were inferred using the Neighbor-Joining method.

**Figure 4:**
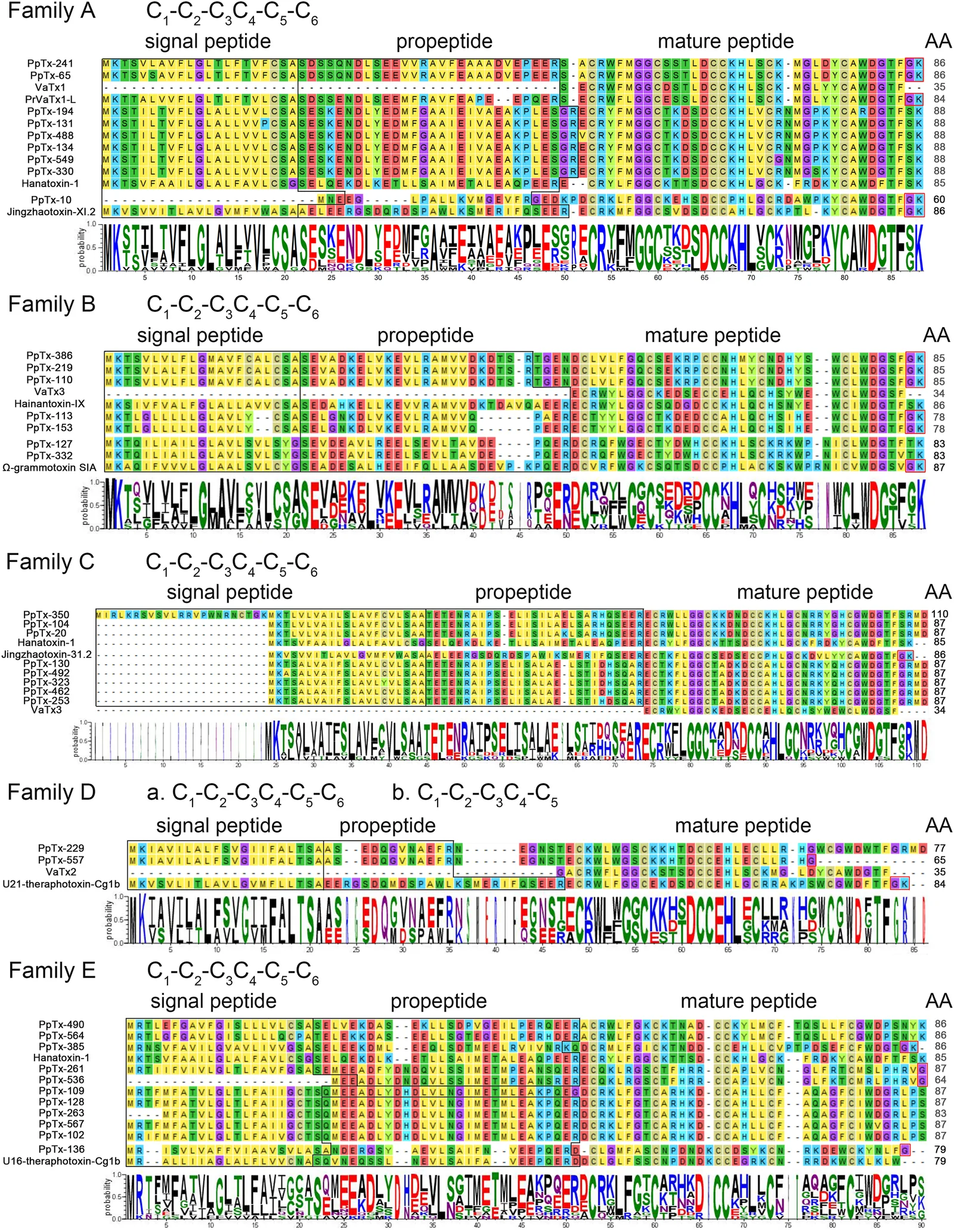

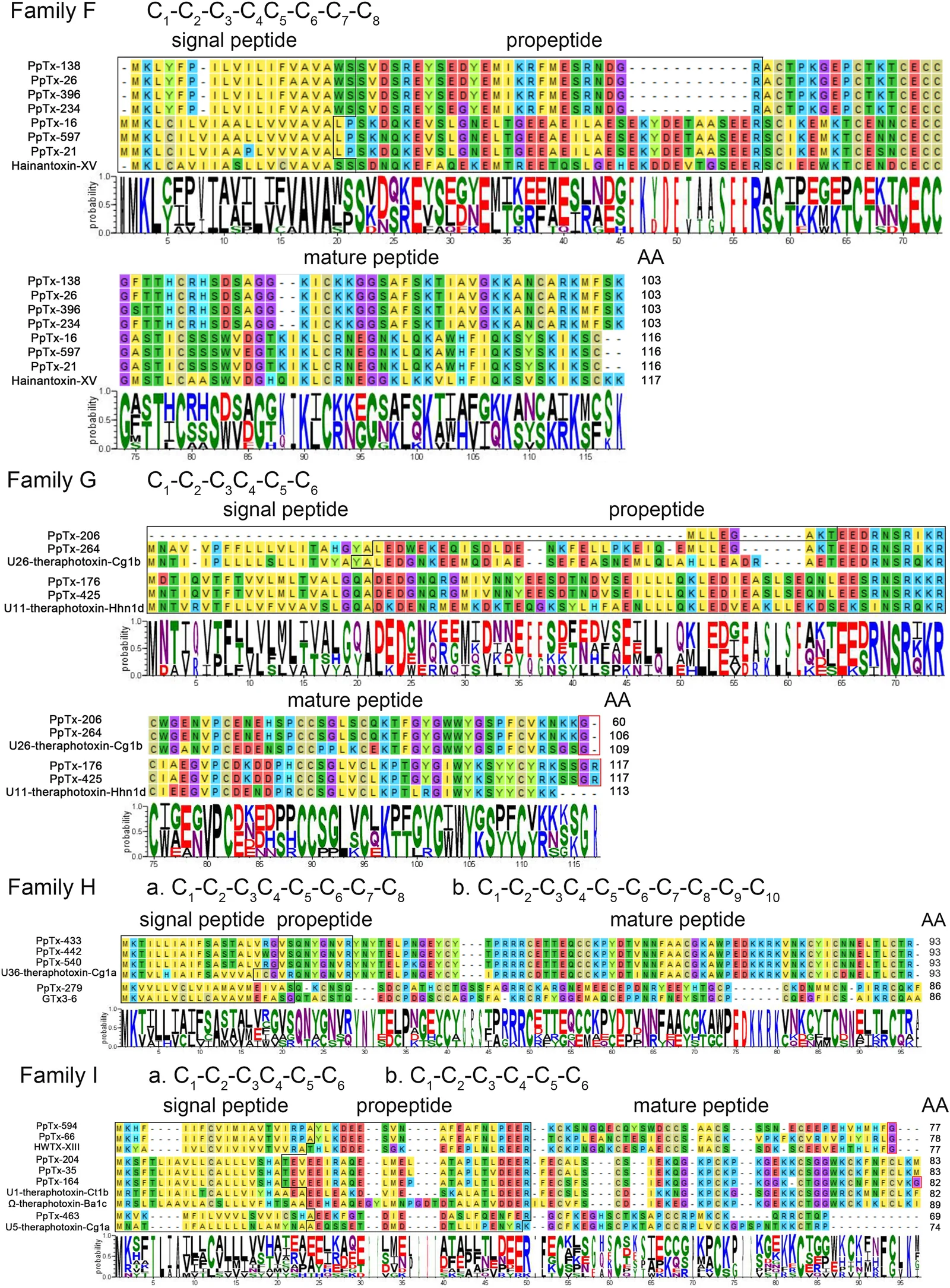

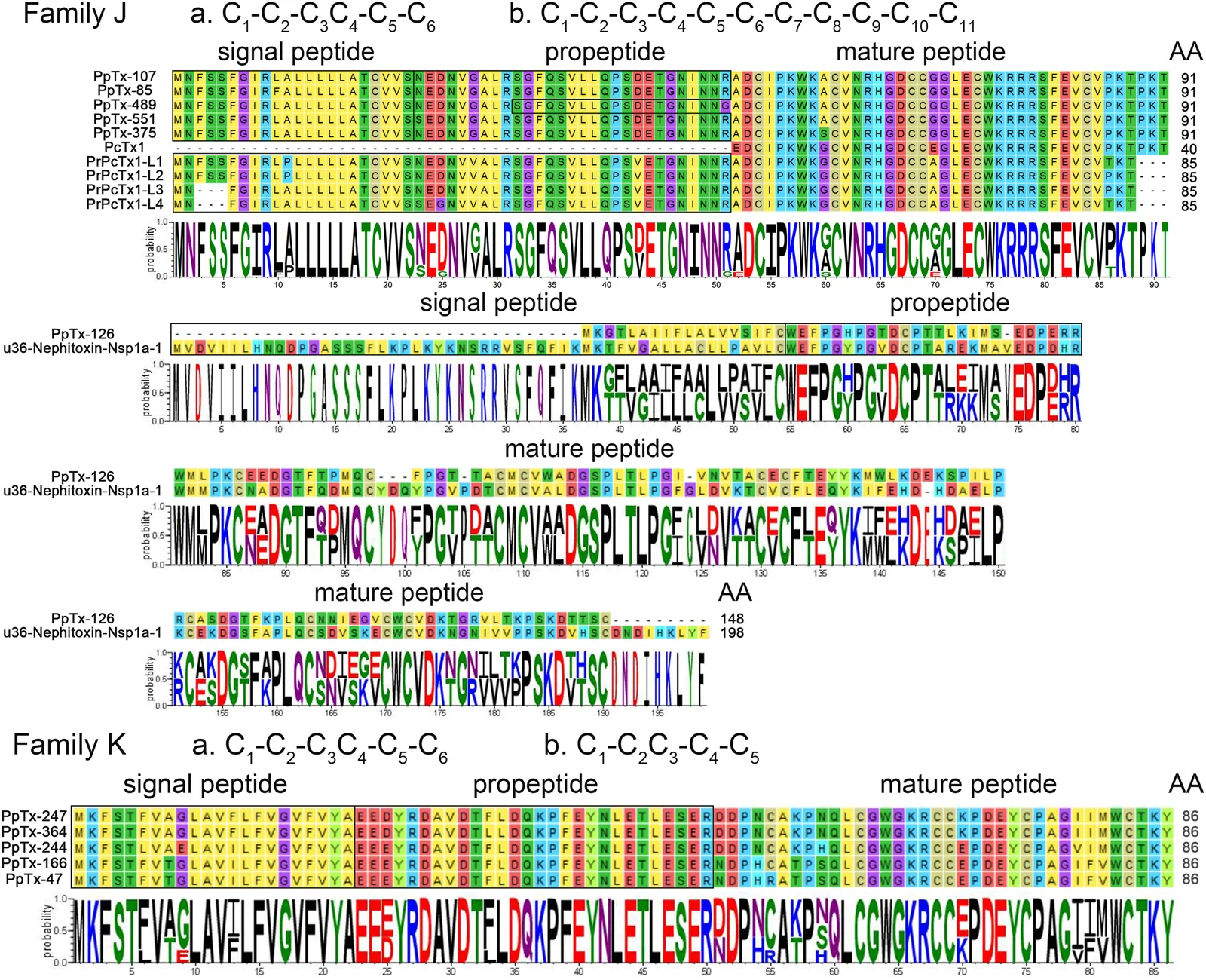
**Multiple sequence alignment of putative toxin precursors**. Toxin precursors from superfamilies *A*–*K* identified in the *P. pulcher* venom-gland cDNA library are shown**.***Black boxes* indicate the signal peptide and propeptide regions, the *red-highlighted box* denotes the C-terminal amidation signal, and the remaining sequences correspond to the predicted mature peptides. Amino acid frequency distributions and consensus motifs are shown beneath each alignment to facilitate visualization of residue conservation.

In addition to the cross-species comparison, we further compared the transcriptomic dataset with MS signals obtained from crude spider venom. Theoretical molecular weights of mature peptides for 42.0% of these NR precursors matched observed MS peaks, suggesting a possible partial overlap between the constructed cDNA library and the preliminarily isolated peptide toxins (Fig. 1*B*). These sequences were aligned and used to infer a phylogenetic tree using Neighbor Joining method, which uncovered a clear clustering pattern that reflects their evolutionary divergence (Fig. 3). Based on sequence similarity and conserved cysteine frameworks, all precursors were classified into 11 distinct families (Families A–K), as shown in Figure 4. This classification captures both lineage-specific expansions and deep evolutionary relationships among toxin genes. All identified precursors belong to the cysteine-rich peptide superfamily, a dominant structural class in venomous animals characterized by compact scaffolds and multiple disulfide bonds. To standardize toxin nomenclature, each precursor was designated as PpTx-n, where Pp denotes *P. pulcher*, Tx indicates toxin, and n corresponds to the clone number.

Building upon this classification, we further examined the structural and functional features of each family. The 3D structures of mature peptides derived from all precursors were predicted using AlphaFold 3, and potential biological functions were explored with reference to BLAST searches against the NR database. The detailed analyses of each toxin family are presented in the following sections.

#### Family A

**Figure 5:**
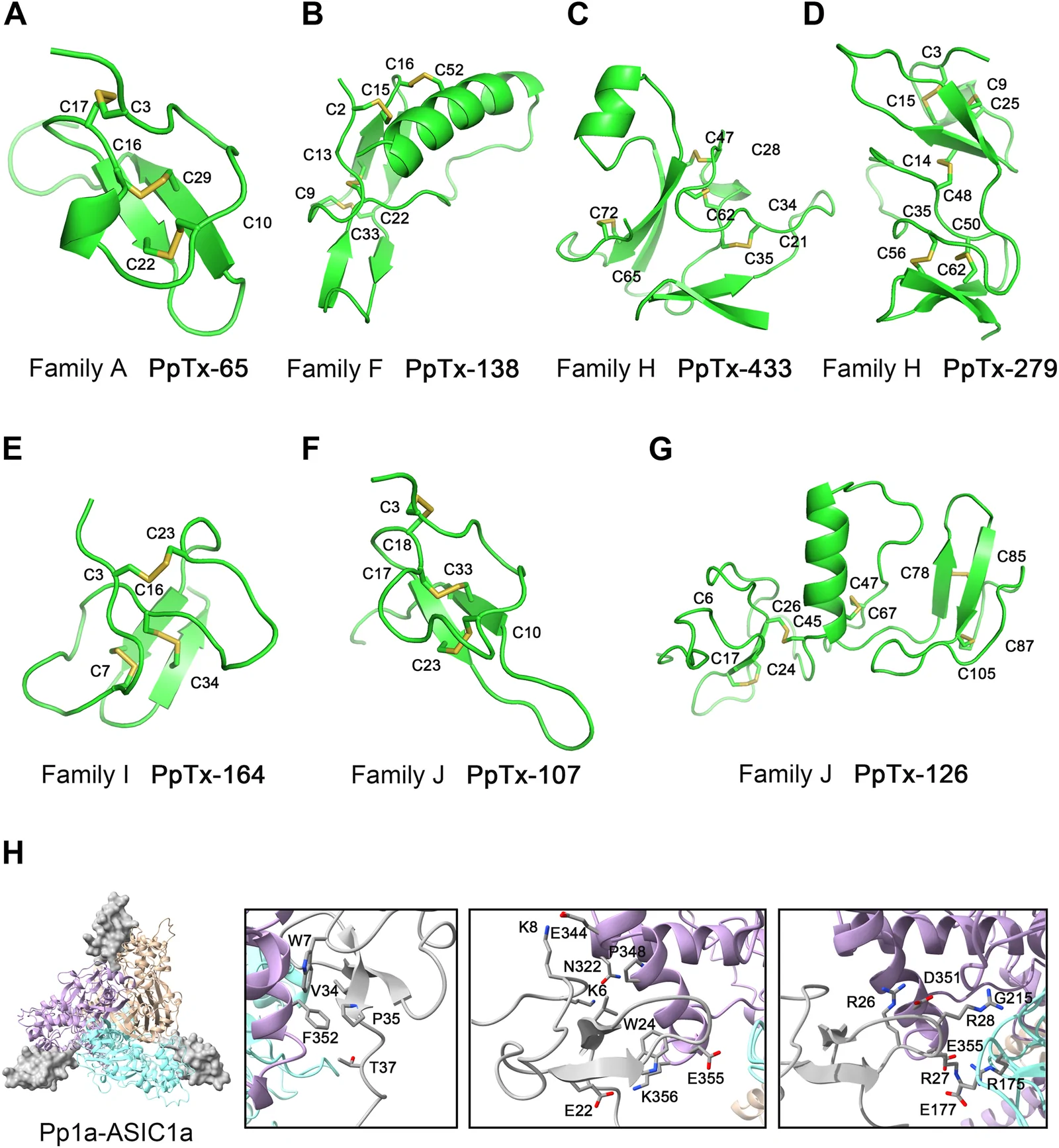
**Predicted 3D structures of representative mature peptides derived from selected toxin precursors and molecular docking analysis of Pp1a with human ASIC1a.***A*–*G*, show the predicted structures of the mature peptides corresponding to PpTx-65, PpTx-138, PpTx-433, PpTx-279, PpTx-164, Pp1a, and PpTx-126, respectively. Family numbers shown beside each peptide. Disulfide bonds are highlighted in *yellow*. *H*, predicted structure of the Pp1a–ASIC1a complex generated by AlphaFold 3 (iptm = 0.79, ptm = 0.80, ranking score = 0.86). Pp1a is shown in *dark gray*, while ASIC1a chain A and chain B are colored *purple* and *light blue*, respectively. Key interacting residues are displayed with side chains and labeled for clarity. The docking model is shown from three different orientations to facilitate visualization of the interaction interface. ASIC1a, acid-sensing ion channel 1a.

Family A comprises nine toxin precursors, all of which contain a signal peptide followed by a propeptide region. All predicted mature peptides contain a conserved cysteine framework C_1_–C_2_–C_3_C_4_–C_5_–C_6_, with a predicted disulfide connectivity of C_1_-C_4_, C_2_-C_5_, and C_3_-C_6_, characteristic of inhibitory cystine knot (ICK) toxins, as exemplified by the predicted structure of PpTx-65 (Fig. 5*A*). In addition, PpTx-10, PpTx-65, and PpTx-241 harbor a C-terminal amidation signal (GK), suggesting posttranslational amidation. Based on sequence homology, Family A can be subdivided into two groups. The first group includes eight precursors (PpTx-241, PpTx-65, PpTx-194, PpTx-131, PpTx-488, PpTx-134, PpTx-549, and PpTx-330), each comprising 86 to 88 amino acid residues. These precursors display high sequence conservation in their signal peptides and propeptide regions, including conserved propeptide cleavage motifs (*e.g.*, PEER or ESGR), indicating strong evolutionary conservation of secretion and processing elements. BLAST analysis revealed moderate sequence identity (57–65%) with Hanatoxin-1 from the Chilean tarantula *Grammostola rosea*, a well-characterized gating modifier of the Kv2.1 potassium channel (20, 21), and moderate sequence identity (65–86%) to VaTx1 from *P. cambridgei*, a congeneric species. Among these peptides, the mature peptide of PpTx-65 differs from VaTx1 by only three amino acid residues, and shows only five amino acid differences relative to the predicted mature peptide of the VaTx1-like precursor (designated PrVaTx1-L in Fig. 4) identified in *P. reduncus* (16). VaTx1 has been reported to activate the capsaicin receptor TRPV1 and induce inflammatory pain (9); however, the amino acid residues responsible for its activity have not been identified, and therefore it remains uncertain whether VaTx1-like toxins in *Psalmopoeus* retain similar functional properties. In contrast, PpTx-10 constitutes a distinct subgroup. Its precursor is markedly shorter than those of other Family A members, and its signal peptide consists of only three residues, which is unusually short for spider toxin precursors and likely results from a gene mutation. PpTx-10 shares moderate sequence identity (60%) with Jingzhaotoxin-XI.2 (also named κ-theraphotoxin-Cg1a-2) from *Chilobrachys jingzhao*, a gating modifier toxin that inhibits Kv2.1 channels and sodium currents in rat cardiac myocytes, which predominantly express Nav1.5 channel (22). This moderate level of sequence conservation indicates that PpTx-10 could potentially possesses comparable ion-channel modulatory activity. Overall, the conserved cysteine framework and sequence homology indicate that Family A peptides constitute a distinct group of spider toxins with putative ion-channel modulatory roles.

#### Family B

Family B comprises seven toxin precursor sequences, each containing a signal peptide and a propeptide region. The precursors are 78 to 85 residues in length. All predicted mature peptides contain a conserved C_1_–C_2_–C_3_C_4_–C_5_–C_6_ framework, with a disulfide connectivity identical to that of the ICK toxins in Family A. Based on sequence homology, Family B can be subdivided into two groups (1): PpTx-386, PpTx-219, PpTx-110, PpTx-113, and PpTx-153 share 38 to 69% sequence identity with Hainantoxin-IX (also named Ω-theraphotoxin-Hhn1f1) from the venom of the spider *Cyriopagopus hainanus*. All mature peptides in this group contain a conserved C-terminal glycine–lysine (GK) motif, indicative of C-terminal amidation *via* posttranslational processing. Hainantoxin-IX has been reported to reversibly block N-type and P/Q-type voltage-gated calcium (Cav) channels in rat dorsal root ganglion neurons, as well as Cav channels in cockroach dorsal unpaired median neurons (23), which may indicate that related peptides could exhibit similar activity. In addition, these peptides show approximately 42 to 71% sequence identity with VaTx3, a toxin from *P. cambridgei* that specifically activates TRPV1 without affecting Kv2.1, unlike VaTx1 and VaTx2 (9). (2) PpTx-127 and PpTx-332 lack a C-terminal amidation site and differ only by a single C-terminal residue (Phe in PpTx-127 *versus* Val in PpTx-332). Both share approximately 57% identity with Ω-grammotoxin SIA (also named Ω-theraphotoxin-Gr1a) from *G. rosea*, a reported blocker of Cav2.1 and Cav2.2 channels, raising the possibility of comparable potential for calcium-channel inhibition (24). Collectively, Family B peptides exhibit homology to toxins acting on diverse ion channels, indicating that sequence-based inference may serve only as a preliminary guide for subsequent functional studies.

#### Family C

Family C consists of eight toxin precursor sequences, each containing a signal peptide and a propeptide region with a predicted proteolytic cleavage site (SEER or SQAR) according to the Processing Quadruplet Motif model. All predicted mature peptides contain six cysteine residues conserved at homologous positions and arranged in the characteristic C_1_–C_2_–C_3_C_4_–C_5_–C_6_ framework, with a predicted disulfide-bonding pattern consistent with the ICK motif. Although PpTx-350 possesses a relatively longer signal peptide, the regions of signal peptide and propeptide within this family remain highly conserved overall. Sequence homology analysis revealed that PpTx-20, PpTx-104, and PpTx-350 show the identical mature peptide, and share relatively moderate sequence identity (61%) with Hanatoxin-1. Other members of this family, including PpTx-130, PpTx-253, PpTx-323, PpTx-462, and PpTx-492, exhibit approximately 58% sequence identity with Jingzhaotoxin-31.2 (encoding Jingzhaotoxin-IV) from the spider venom *C. jingzhao*. Jingzhaotoxin-IV has been reported to specifically inhibit Nav1.5 channel by modulating activation and inactivation, *via* a mechanism distinct from classical site three and site four sodium channel toxins (25). These results indicate that Family C peptides may target voltage-gated ion channels, although their precise channel modulation remains to be experimentally validated. Furthermore, the sequence identity between these peptides and VaTx3 is approximately 45%. Collectively, these sequence and structural features indicate that Family C constitutes a conserved group of cysteine-rich peptides with an ICK scaffold and potential ion channel–modulatory activity, warranting further functional investigation.

#### Family D

Family D comprises only two precursors, PpTx-229 and PpTx-557. Sequence analysis revealed that these precursors share identical signal peptide and propeptide regions, consisting of 21 and 12 residues, respectively. Comparison of the mature peptides showed that their N-terminal 32 residues are completely identical; however, PpTx-557 lacks the final 12 C-terminal residues present in PpTx-229. This truncation is likely caused by premature translational termination resulting from a nucleotide mutation, in which the tryptophan codon (TGG) is converted into a stop codon (TGA). The mature peptide of PpTx-229 contains six cysteine residues arranged in the conserved C_1_–C_2_–C_3_C_4_–C_5_–C_6_ framework, with a predicted disulfide connectivity of C_1_–C_4_, C_2_–C_5_, and C_3_–C_6_. This connectivity positions the extended N- and C-terminal regions on opposite sides of the toxin scaffold. In contrast, due to premature termination, the mature peptide of PpTx-557 contains only five cysteine residues. Disulfide bond prediction indicates that PpTx-557 can form only two disulfide bonds (C_1_–C_4_ and C_2_–C_5_), leaving C_3_ unpaired, which is expected to markedly disrupt peptide folding and biological function. Both PpTx-229 and PpTx-557 share approximately 45 to 48% sequence identity with U21-theraphotoxin-Cg1b (Jingzhaotoxin-39) from the spider *C*. *jingzhao* (26), whose biological function has not yet been characterized. In addition, PpTx-229 exhibits a low level of sequence similarity (39%) to VaTx2; however, this degree of homology alone is insufficient to infer a shared TRPV1-targeting activity, and no functional implication can be drawn at present.

#### Family E

Family E comprises 11 toxin precursors. Although all predicted mature peptides contain six cysteine residues arranged in the conserved C_1_–C_2_–C_3_C_4_–C_5_–C_6_ framework, with a predicted disulfide connectivity of C_1_-C_4_, C_2_-C_5_, and C_3_-C_6_, the precursor sequences exhibit substantial variability and can be subdivided into two groups based on sequence divergence. (1) 10 closely related precursors (PpTx-490, PpTx-564, PpTx-385, PpTx-261, PpTx-536, PpTx-109, PpTx-128, PpTx-263, PpTx-567, and PpTx-102). Among them, PpTx-536 contains an unusually short signal peptide of only three residues, markedly shorter than those of other family members, likely resulting from a mutation at the translation initiation site leading to N-terminal truncation. In PpTx-109, substitution of the terminal arginine residue in the propeptide with glycine abolishes the proteolytic cleavage site, yielding a precursor lacking a propeptide region and resulting in the formation of an N-terminal α-helical segment. In addition, the mature peptides of PpTx-385, PpTx-261, and PpTx-536 harbor a C-terminal amidation signal (“GK” or “G”), suggesting posttranslational amidation. The mature peptides of PpTx-128, PpTx-263, PpTx-567, and PpTx-102 are either identical or differ by only one residue. Sequence alignment revealed that peptides in this group share relatively low sequence identity (22–49%) with Hanatoxin-1, and functional relatedness cannot be reliably inferred from sequence alone. (2) In contrast to the other 10 members, the mature peptide of PpTx-136 displays pronounced sequence divergence. It shares 68% sequence identity with U16-theraphotoxin-Cg1b (Jingzhaotoxin-26) from *C*. *jingzhao*, a toxin whose biological function has not yet been reported (26). This marked divergence suggests that PpTx-136 represent a distinct evolutionary lineage within Family E. Taken together, these observations support the classification of Family E as a structurally conserved yet sequence-diverse group of ICK-type spider toxins.

#### Family F

Family F comprises seven precursor peptides, each containing a signal peptide and a propeptide region. In contrast to the toxins described in the preceding families, the predicted mature peptides of Family F are relatively long, consisting of 59 amino acid residues, with theoretical molecular masses ranging from approximately 6.0 to 6.7 kDa. All mature peptides contain a conserved linear cysteine framework (C_1_–C_2_–C_3_–C_4_C_5_–C_6_–C_7_–C_8_); however, predicted disulfide bond connectivity, determined *via* AlphaFold 3, exhibit notable diversity among family members. Specifically, PpTx-138, PpTx-26, PpTx-396, and PpTx-234 are predicted to form four disulfide bonds with the connectivity C_1_-C_4_, C_2_-C_6_, C_3_-C_7_, and C_5_-C_8_, and share low sequence identity (∼31%) with Hainantoxin-XV from *C*. *hainanus* (the predicted 3D structure of the mature peptide of PpTx-138 is shown in Fig. 5*B*). In contrast, PpTx-16, PpTx-597, and PpTx-21 are predicted to adopt an alternative disulfide connectivity pattern (C_1_-C_5_, C_2_-C_6_, C_3_-C_8_, and C_4_-C_7_) and share relatively high sequence identity (75–76%) with Hainantoxin-XV, for which no biological activity has yet been reported. Despite the observed sequence similarities, the marked differences in predicted disulfide connectivity suggest potential structural divergence, and the precise biological functions of these peptides therefore remain to be elucidated.

#### Family G

Family G comprises four toxin precursors. All predicted mature peptides contain six cysteine residues at homologous positions, arranged in the conserved C_1_–C_2_–C_3_C_4_–C_5_–C_6_ framework, with a predicted disulfide connectivity of C_1_-C_4_, C_2_-C_5_, and C_3_-C_6_. All mature peptides also possess a C-terminal amidation signal (“G” or “GR”). Based on sequence similarity, the four precursors can be subdivided into two groups: (1) PpTx-206 and PpTx-264. PpTx-206 has a signal peptide and propeptide of 8 and 10 residues, respectively, markedly shorter than those of PpTx-264 (22 and 44 residues). Both share identical mature peptide and relatively high sequence identity (73%) with U26-theraphotoxin-Cg1b (Jingzhaotoxin-57) from the spider *C*. *jingzhao*, a toxin with unknown function (26). (2) PpTx-176 and PpTx-425. These two precursors differ only at the second residue of the N terminus and exhibit 78% sequence identity with U11-theraphotoxin-Hhn1d from *C*. *hainanus*, another toxin with an uncharacterized biological function (23). In summary, the functional properties of Family G members remain to be experimentally investigated.

#### Family H

Family H comprises four precursors. Based on sequence homology, the family can be divided into two subgroups: (1) PpTx-433, PpTx-442, and PpTx-540, whose predicted mature peptides share an eight-cysteine framework (C_1_–C_2_–C_3_C_4_–C_5_–C_6_–C_7_–C_8_) with a predicted disulfide connectivity of C_1_-C_4_, C_2_-C_5_, C_3_-C_6_, and C_7_-C_8_ (as illustrated for the mature peptide of PpTx-433 in Fig. 5*C*). Multiple sequence alignment reveals a high degree of conservation among their mature peptides, with PpTx-433 and PpTx-442 being identical and PpTx-540 differing by only a single residue. These peptides exhibit high sequence identity (92–94%) to U36-theraphotoxin-Cg1a from the spider *C. jingzhao* (26); however, as the biological function of this reference toxin has not yet been characterized, functional inference for this subgroup remains limited. (2) PpTx-279 lacks a propeptide region, suggesting a distinct precursor architecture. Its predicted mature peptide displays an expanded ten-cysteine framework (C_1_–C_2_–C_3_C_4_–C_5_–C_6_–C_7_–C_8_–C_9_–C_10_) with a predicted disulfide bonding pattern of C_1_-C_4_, C_2_-C_5_, C_3_-C_7_, C_6_-C_9_, and C_8_-C_10_ (predicted structure shown in Fig. 5*D*). The increased number of cysteine residues implies a more densely cross-linked structure, which may stabilize a distinct three-dimensional fold. PpTx-279 shows relatively moderate sequence identity (approximately 52%) to GTx3-6 from *G. rosea* (Accession number: BAN13526), whose biological function has not been experimentally validated.

#### Family I

Family I comprises six toxin precursors, all of which contain both a signal peptide and a propeptide. Based on sequence homology and cysteine framework architecture, these precursors can be subdivided into three subgroups. (1) PpTx-66 and PpTx-594 are characterized by a cysteine framework of C_1_–C_2_–C_3_C_4_–C_5_–C_6_ and a conserved C-terminal amidation signal (G). The predicted disulfide connectivity follows the canonical pattern of C_1_–C_4_, C_2_–C_5_, and C_3_–C_6_. This structural organization is consistent with that of their closest homolog, HWTX-XIII, with which they share approximately 32 to 61% identity; however, the biological activity of HWTX-XIII has not been reported (27). (2) PpTx-204, PpTx-35, and PpTx-164 exhibit a linear cysteine arrangement of C_1_–C_2_–C_3_–C_4_–C_5_–C_6_ in their predicted mature peptides. Structural prediction suggests that these peptides adopt a fold similar to that of HWTX-II and contain a disulfide-directed hairpin (DDH) motif, with a predicted disulfide bonding pattern of C_1_-C_3_, C_2_-C_5_, and C_4_-C_6_ (predicted structure of PpTx-164 mature peptide shown in Fig. 5*E*) (28). Notably, among this subgroup, only PpTx-164 possesses a clear C-terminal amidation signal, indicating potential heterogeneity in posttranslational modification. Members of this subgroup share approximately 67 to 81% sequence identity with U1-theraphotoxin-Ct1b from the Australian theraphosid spider *Coremiocnemis tropix*, a toxin reported to be lethal to the sheep blowfly *Lucilia cuprina* (29). In addition, they exhibit about 68 to 74% identity with Ω-theraphotoxin-Ba1c from the spider *Brachypelma albiceps*, which has been shown to enhance the insecticidal efficacy of recombinant baculoviruses (30). Although both toxins have been reported to exhibit insecticidal effects, their precise molecular targets and action mechanisms remain unknown, yet this subgroup may still hold potential for biological insecticide development. (3) PpTx-463 shares the compact C_1_–C_2_–C_3_C_4_–C_5_–C_6_ cysteine framework characteristic of subgroup one, with a predicted disulfide connectivity of C_1_-C_4_, C_2_-C_5_, and C_3_-C_6_. It shares approximately 72% sequence identity with U5-theraphotoxin-Cg1a from *C*. *jingzhao* (26); however, its biological function remains unclear and will require further functional characterization. Collectively, the structural diversity and homology to known insecticidal toxins could constitute a promising source of bioactive molecules, pending further functional validation.

#### Family J

Family J consists of six toxin precursors characterized by a conserved signal peptide and propeptide architecture, which can be further divided into two subgroups based on sequence homology. (1) PpTx-107, PpTx-85, PpTx-489, PpTx-551, and PpTx-375 show sequence identity ranging from 62% to 93% with PcTx1, a well-characterized ASIC1a inhibitor from the congeneric species *P*. *cambridgei* (11, 12), suggesting that their predicted mature peptides may share related biological activities. Notably, the predicted mature peptide sequences of PpTx-107, PpTx-85, and PpTx-551 are identical and differ from PcTx1 by only three amino acid residues, none of whose homologous positions have been examined in previous alanine-scanning mutagenesis studies of PcTx1 or implicated in the PcTx1-ASIC1a interaction network (31, 32, 33). Nevertheless, deletion of the first two N-terminal residues in PcTx1 (ΔN2-PcTx1) resulted in an approximately 7-fold reduction in inhibitory activity against human ASIC1a, while showing no significant reduction in inhibitory activity toward rat ASIC1a (33). However, whether the substitution at the first N-terminal residue contributes to inhibitory activity remains unclear. Further comparison with PcTx1-like peptides compiled from *P. reduncus* (abbreviated as PrPcTx1-L in Fig. 4) revealed that sequence divergence is primarily concentrated at the C terminus, particularly in the loss of the repeated PKT motif. Although several C-terminal residues have been implicated in toxin–channel interactions, previous studies suggest that truncation of this region exerts relatively limited effects on channel modulation (32, 33). Collectively, these findings indicate that PcTx1-like precursor in *P. reduncus* are highly conserved within the genus *Psalmopoeus*, although the evolutionary significance and physiological role of this conserved toxin group in spiders remain unclear. In contrast, PpTx-489 contains a mutation at the propeptide cleavage site, resulting in an N-terminal extension of the mature peptide, whereas PpTx-375 differs from the consensus sequence by only a single residue substitution. All members’ mature peptides of this subgroup retain the canonical C_1_–C_2_–C_3_C_4_–C_5_–C_6_ cysteine framework, and the disulfide connectivity is predicted to follow the conserved pattern of C_1_-C_4_, C_2_-C_5_, and C_3_-C_6_ (predicted structure of the mature peptide of PpTx-107 shown in Fig. 5*F*).

(2) PpTx-126 is the longest precursor identified in this study, comprising 148 amino acid residues, and its predicted mature peptide has a molecular mass of approximately 11.7 kDa. The sequence is highly divergent and shares only moderate sequence identity (55%) with U36-Nephitoxin-Nsp1a-1 (accession number: GIY60125.1) from the evolutionarily distant araneid spider *Caerostris darwini* (34); however, the biological function of this toxin remains uncharacterized. The mature peptide of PpTx-126 exhibits an expanded cysteine framework (C_1_–C_2_–C_3_–C_4_–C_5_–C_6_–C_7_–C_8_–C_9_–C_10_–C_11_). Based on structural prediction of the mature peptide, the disulfide bonding pattern is proposed to be C_2_-C_3_, C_4_-C_5_, C_6_-C_7_, C_8_-C_9_, and C_10_-C_11_, with C_1_ remaining unpaired. In the predicted structural model, an α-helix effectively separates the N-terminal and C-terminal regions into two independent domains, whereas the C-terminal domain is stabilized by three disulfide bonds (predicted structure shown in Fig. 5*G*). Whether this unusual architecture, characterized by an expanded cysteine framework and domain-like organization, confers broader or more complex biological activities remains to be determined by future functional studies. The coexistence of a well-conserved ASIC1a-related subgroup and a highly divergent, multidomain-like peptide underscores the functional and structural diversity of Family J and warrants further experimental investigation.

#### Family K

Family K comprises five toxin precursor sequences, namely PpTx-247, PpTx-364, PpTx-244, PpTx-166, and PpTx-47, all of which contain a canonical signal peptide and a propeptide region. Multiple sequence alignment revealed a high degree of sequence conservation within this family. Notably, PpTx-247 and PpTx-364 encode identical mature peptide sequences. In addition, PpTx-166 and PpTx-47 share identical signal peptide and propeptide regions. Compared with PpTx-166, the mature peptide of PpTx-47 exhibits a distinctive variation at the position corresponding to the first cysteine residue. In PpTx-47, this cysteine is replaced by a basic arginine residue, resulting in a reduced cysteine framework of C_1_–C_2_C_3_–C_4_–C_5_. Accordingly, the disulfide connectivity of PpTx-47 is predicted to be C_1_-C_4_ and C_2_-C_5_. In contrast, the remaining family members retain the canonical C_1_–C_2_–C_3_C_4_–C_5_–C_6_ cysteine framework, with a predicted disulfide bonding pattern of C_1_-C_4_, C_2_-C_5_, and C_3_-C_6_. Despite the high level of sequence conservation within Family K, no closely related sequences were identified in public databases, indicating that this family may represent a previously unreported group of spider venom peptides. Family K represents a highly conserved yet previously unrecognized group of spider venom peptides, featuring subtle but potentially functionally significant variations in cysteine composition and disulfide connectivity.

### 3D structures of representative peptide toxins and their molecular docking with ion channels

The mature peptides derived from all predicted toxin precursors were structurally modeled using AlphaFold 3. Only representative examples are shown in Figure 5 to illustrate structural diversity and functional relevance. Many families share similar cysteine frameworks and predicted disulfide connectivities, resulting in highly similar overall structures; for example, members of Families A, B, C, E, and G are predicted to adopt comparable folds. As shown in Figure 5, *A*–*G*, PpTx-65 (VaTx1-like toxin) and PpTx-107 (PcTx1-like toxin) represent canonical ICK-type scaffolds (Fig. 5, *A* and *F*), whereas PpTx-164 adopts DDH-type scaffolds (Fig. 5*E*). Peptides with more extensive disulfide networks, including PpTx-138, PpTx-433, and PpTx-279, display alternative cysteine connectivity and potentially denser cross-linking (Fig. 5, *B*–*D*). PpTx-126 features a predicted α-helical segment that separates the N- and C-terminal regions into two relatively independent domains (Fig. 5*G*). Collectively, these representative structures illustrate the structural diversity of *P. pulcher* venom peptide in terms of disulfide connectivity, folding patterns, and domain organization.

To assess potential functional relevance, molecular docking analyses were performed to evaluate the interactions between representative peptides and ion channels inferred from homology-based predictions. In particular, the mature peptide encoded by PpTx-107, PpTx-85, and PpTx-551 was selected to predict the complex structure with human ASIC1a channel. Based on its predicted interaction with ASIC1a and subsequent functional validation, this mature peptide has been renamed according to the rational toxin nomenclature as π-theraphotoxin-Pp1a (abbreviated as Pp1a) to avoid ambiguity (35). The complex model of three Pp1a molecules and ASIC1a trimer is as demonstrated in Figure 5*H*. Analysis of the predicted the interface of channel with a peptide revealed an extensive network of electrostatic and hydrophobic interactions stabilizing the peptide-channel interface (Table 1). In the predicted model, the N terminus of Pp1a is positioned at the periphery of the peptide-channel interface. Therefore, the structural basis underlying the effect of truncating the first two N-terminal residues of PcTx1 on human ASIC1a inhibitory activity remains unclear and is not addressed by the current data. Multiple lysine residues of Pp1a, including Lys8, Lys26, and Lys27, formed salt bridges with acidic residues on a subunit (chain A) of ASIC1a channel, notably Glu344, Asp351, and Glu355. In addition, Arg27 also established a salt bridge with Glu177 on the neighboring subunit (chain B), while a reciprocal salt bridge was identified between peptide Glu22 and Lys356 on chain B. Hydrogen bonds were observed between Lys6 and Asn322, as well as between Arg28 and Gly215 on chain B. Moreover, hydrophobic interactions further contributed to complex stabilization. Trp7, Trp24, Val32, Trp34, Pro35, and Thr37 collectively formed a large hydrophobic interaction interface with the hydrophobic side-chain regions of Phe352, Glu355, Lys356, and Pro348 on chain A, as well as Arg175 and Glu177 on chain B. Notably, Phe352 in the present structure corresponds to Phe350 in rat ASIC1a due to residue numbering differences. These interactions suggest a plausible binding mode driven primarily by electrostatic forces and reinforced by hydrophobic contacts, closely resembling the molecular mechanism underlying PcTx1-mediated inhibition of ASIC1a (32).

**Table 1:** Contact analysis of the Pp1a-human ASIC1a docking models produced by AlphaFold 3

ASIC1a channel	Pp1a	Distance (Å)	Type of contacts
Chain A, Asn322 [OD1]	Lys6 [NZ]	2.482	Hydrogen bond
Chain A, Glu344 [OE2]	Lys8 [NZ]	2.475	Salt bridge
Chain A, Asp351 [OD2]	Arg26 [NE]	2.568	Salt bridge
Chain A, Asp351 [OD2]	Arg26 [NH2]	2.864	Salt bridge
Chain A, Glu355 [OE1]	Arg27 [NE]	2.87	Salt bridge
Chain B, Glu177 [OE2]	Arg27 [NH1]	3.168	Salt bridge
Chain B, Gly215 [O]	Arg28 [NH2]	2.059	Hydrogen bond
Chain A, Lys356 [NZ]	Glu22 [OE1]	2.506	Salt bridge
Chain A, Phe352	Trp7, Val34, Pro35, Thr37	-	Hydrophobic interaction
Chain A, Glu355, Lys356	Trp24	-	Hydrophobic interaction
Chain A, Pro348	Val32	-	Hydrophobic interaction
Chain B, Arg175, Glu177	Arg27	-	Hydrophobic interaction

Subsequently, molecular docking was performed on the highly divergent mature peptides derived from Family K precursors to explore their potential ion-channel targets. Due to computational limitations, only a subset of well-characterized channels was screened, including the voltage-gated ion channels Kv1.3, Cav2.2, and Nav1.2, as well as the inwardly rectifying potassium (Kir) channels Kir1.1, Kir4.1, and Kir6.2. As summarized in Table S1, Family K peptides showed low-confidence interactions with Cav2.2 and Nav1.2, whereas higher-confidence complexes were obtained for Kv1.3 and several Kir channels. As shown in Fig. S2, the representative PpTx-247 was positioned along the lateral surface of the Kv1.3 voltage-sensing domain, while the PpTx-247–Kir6.2 complex placed the peptide near the outer pore region. Notably, neither binding mode appeared to involve the basic side chains typically associated with pore-blocking toxins, and therefore these predicted poses merit further functional investigation. These docking results provide preliminary insights into the potential targets and interaction modes of Family K peptides. Taken together, molecular docking provided preliminary structural information for both the conserved ASIC1a-targeting peptide Pp1a and the highly divergent Family K peptides, serving as a basis for future experimental evaluation of their potential ion channel targets and interaction modes.

### Identification of Pp1a and its inhibitory activity against ASIC1a

**Figure 6:**
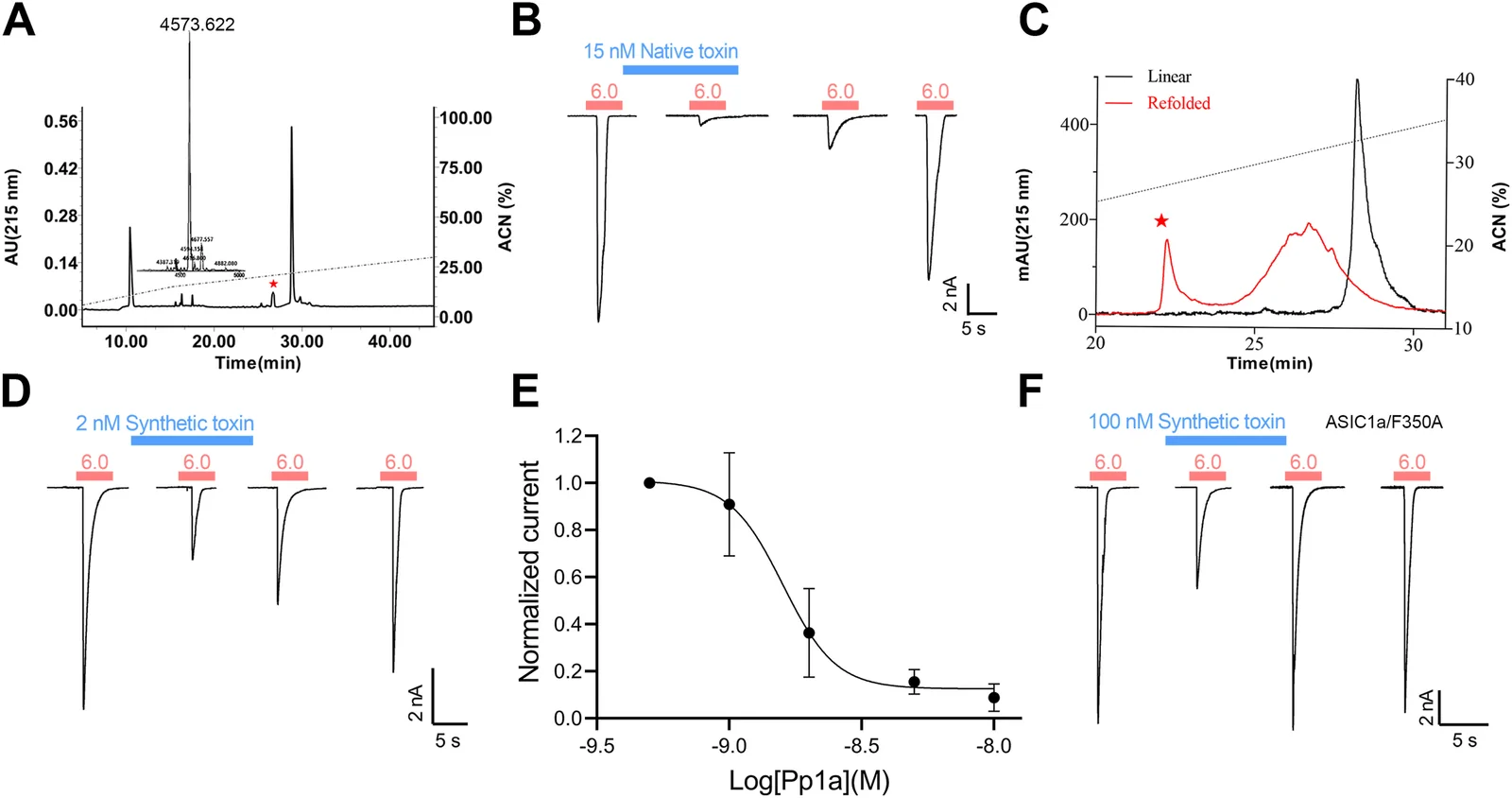
**Identification of Pp1a and its inhibitory effects on rat ASIC1a.***A*, purification of the chromatographic fraction containing Pp1a by RP-HPLC. The *red star* indicates the peak corresponding to Pp1a. The inset shows the MALDI-TOF mass spectrum of this fraction. *B*, representative current traces showing inhibition of ASIC1a currents by 15 nM native Pp1a. *C*, RP-HPLC purification profiles of the chemically synthesized linear Pp1a and the refolded peptide. The *red star* marks the correctly oxidatively folded Pp1a. *D*, representative current traces showing inhibition of ASIC1a current by 2 nM synthetic Pp1a. *E*, concentration-response relationship for inhibition of ASIC1a currents by synthetic Pp1a. *F*, representative current traces showing the markedly reduced inhibitory effect of synthetic Pp1a at a high concentration (100 nM) on the ASIC1a/F350A mutant channel. In panels *B*, *D*, and *F*, ASIC1a currents were evoked by brief acidic application, with a 60 s bath flow between stimuli. Toxin was applied by continuous bath flow for 3 min and maintained during stimulation. ASIC1a, acid-sensing ion channel 1a; MALDI-TOF MS, matrix-assisted laser desorption/ionization time-of-flight mass spectrometry; RP-HPLC, reverse-phase high-performance liquid chromatography.

Homology analysis and molecular docking-based predictions suggested that Pp1a may possess ion channel–modulating activity. To evaluate the consistency between the constructed cDNA library and the native crude venom, and to experimentally assess the functional relevance of the bioinformatic predictions, crude venom fractions were screened for activity against ASIC1a. Screening assays showed that the chromatographic fraction eluting at approximately 22 min, indicated by a red asterisk, exhibited measurable inhibitory activity (Fig. 1*A*); To improve the purity of the initially active fraction, further subfractionation identified the component marked with a red star (26 min in Fig. 6*A*) as the active fraction, and mass spectrometry suggested relatively high purity of this fraction (Fig. 6*A* inset and Fig. S3*A*). Application of the native peptide corresponding to the red-star peak at 15 nM resulted in a 93.8 ± 2.4% inhibition of the current (Fig. 6*B*, n = 5). Based on the comparison between the observed MS signals and the theoretical molecular masses of toxin precursor-derived mature peptides, together with partial Edman degradation sequencing, we inferred that the red-star peak most likely corresponded Pp1a, potentially encoded by PpTx-107, PpTx-85, or PpTx-551. However, the available data do not allow unambiguous discrimination among these ESTs.

To further characterize the biological activity of Pp1a and to circumvent the limited yield of the native venom-derived peptide, a linear synthetic Pp1a was prepared and subjected to oxidative refolding in a GSH/GSSG redox system to reconstitute its native disulfide connectivity, yielding the correctly folded peptide with an overall refolding yield of 6.5% (Fig. 6*C*). Both mass spectrometry analysis and coelution experiments with the native peptide indicated that the refolded peptide was consistent with the native form (Fig. S3, *C* and *D*). Functional assays demonstrated that the refolded peptide produced a potent and reversible inhibition of ASIC1a currents, with an IC_50_ value of 1.8 ± 0.6 nM, confirming that Pp1a is a bioactive venom component targeting ASIC1a (Fig. 6, *D*–*E*, n = 4–6). The Phe350 residue of rat ASIC1a is a critical binding determinant for PcTx1, and alanine substitution at this position has been shown to abolish toxin-mediated inhibition of the channel (36). Consistent with this, even at a high concentration of 100 nM, Pp1a exhibited markedly reduced inhibitory activity against the ASIC1a/F350A mutant channel, producing only 38.7 ± 20.1% inhibition (Fig. 6*F*, n = 3). Collectively, these results suggest that combining homology analysis with molecular docking can facilitate the preliminary identification of candidate bioactive venom peptides for subsequent experimental evaluation.

## Conclusion

Spider venom peptides are indispensable molecular tools for ion-channel research and constitute a valuable reservoir for therapeutic discovery. Toxins from the genus *Psalmopoeus* have contributed significantly to elucidating the structure - function relationships and physiological roles of channels such as ASIC1a and TRPV1. Despite their importance, the toxin gene repertoire of this genus remains largely unexplored. Here, we selected *P*. *pulcher*, a previously uncharacterized species, and examined its venom composition through a combination of venom-gland cDNA library construction and mass spectrometric analysis. Functional exploration of selected characteristic precursors and the highly divergent Family K precursors, using molecular docking and limited experimental validation, provided insight into their potential biological relevance.

Through the cDNA library, we identified 69 NR toxin precursors grouped into 11 families, and mass spectrometry independently revealed 166 distinct mass signals, with a subset of MS signals corresponding to predicted mature peptides from the library, together highlighting the substantial molecular diversity of *P. pulcher* venom. The larger number of MS-detected peptides likely reflect the incomplete coverage of the cDNA library, which focused on higher molecular weight sequences and thus missed many smaller peptides—for instance, 46.2% of detected signals fell below 3000 Da, a range not fully covered by the library. Within families, precursor sequences exhibit recognizable similarity, whereas extensive variation, including substitutions, deletions, and frameshifts, and divergence at otherwise conserved cysteine positions - contributes to the broader transcriptomic diversity, consistent with structural convergence and functional diversification driven by ecological and evolutionary pressures. Structural modeling of the predicted mature peptides revealed pronounced diversity in cysteine connectivity and folding patterns, encompassing canonical ICK and DDH scaffolds, as well as structures with partially missing disulfide bonds due to cysteine substitutions, although these structures remain computational predictions from AlphaFold 3. Notably, PpTx-126 is predicted to adopt a two-domain architecture separated by an α-helical segment. The functional characterization of such multidomain peptides represents a promising and emerging direction in peptide toxin research (37, 38).

The number of toxin precursors identified in *P. pulcher* is comparable to those reported for the tarantulas *G. rosea* and *Ornithoctonus huwena*, but remains lower than that identified in the congeneric species *P. reduncus* using high-throughput sequencing approaches (27, 39), likely related to sequencing depth and transcriptome coverage. Comparative analysis with published *P. cambridgei* sequences and available *P. reduncus* transcriptome entries provide a preliminary indication of genus-level relatedness among these congeners (9, 16). Although toxin precursor peptides from *P. pulcher* and *P. reduncus* exhibit broadly similar amino acid length distributions, overall sequence conservation is relatively low. Interestingly, certain characteristic toxin groups, such as PcTx1-like and VaTx1-like peptides, appear highly conserved across *Psalmopoeus* species. Such functional conservation may contribute to the evolutionary retention of these peptides within the genus. For example, VaTx1-like peptides may induce pain as a defensive mechanism, whereas the physiological role of PcTx1-like peptides in spiders remains less clear. It remains unclear whether these peptides may regulate ASIC1a-related homologs in insect prey, such as members of the pickpocket family (40, 41, 42).

Only a subset of the toxin families (5 of 11) could be tentatively assigned putative functions based on sequence homology, illustrating the limited predictive power of homology-based annotation. Molecular docking analysis indicated that Pp1a adopts a binding mode on ASIC1a highly similar to that of PcTx1. The inhibitory effect of Pp1a on ASIC1a currents was experimentally validated, and the predicted key interacting residues partially overlap with those reported for PcTx1, supporting that Pp1a as a PcTx1-like ASIC1a-modulating toxin within the *Psalmopoeus* genus (12, 32). While a few additional families (*e.g.*, Family A, B, and C) allowed coarse functional inference, most sequences lacked meaningful homology and remain unannotated, reflecting the limited predictive power of homology-based annotation and the incomplete coverage of venom-gland cDNA libraries. To obtain preliminary insights into potential bioactivity, molecular docking was further applied to toxin families lacking informative homology. Due to computational limitations, however, analysis was restricted to the highly divergent Family K precursors, which showed tentative associations with channel such as Kv1.3, Kir1.1, Kir4.1, and Kir6.2. Nevertheless, these predictions require experimental validation. Future high-throughput *in silico* screening may provide a more efficient strategy for systematically profiling mature peptide repertoire, facilitating the discovery of novel ion-channel modulators. Such approaches may also help identify peptides targeting less-characterized ion channels, thereby expanding the repertoire of pharmacological tools and potentially revealing candidates for pest control.

Overall, this study provides a molecular and structural characterization of the venom composition of *P. pulcher*, revealing a diverse repertoire of toxin precursors and pronounced variation in cysteine connectivity and folding patterns. By integrating cDNA library analysis, proteomic evidence, structural modeling, and preliminary functional predictions, we provide a comprehensive resource and reference for further investigation of venom peptides and their potential ion channel–modulating activities. Our findings expand the known toxin landscape of the *Psalmopoeus* genus and highlight the potential of these peptides as molecular probes and candidate modulators of ion channels, including multidomain peptides that represent an emerging direction in venom peptide research.

## Experimental procedures

### Collection, fractionation, and mass spectrometric analysis of *P. pulcher* venom

Adult *P. pulcher* spiders (n = 50; body length 8–12 cm; both sexes) were obtained from a commercial breeder in China and maintained temporarily under laboratory conditions at approximately 25 °C with free access to water and mealworms. Crude venom was obtained by electrical stimulation (5 V), with venom extraction performed at 2-week intervals to allow sufficient time for venom replenishment. The collected venom was pooled, lyophilized, and stored at −20 °C until further use.

Prior to chromatographic separation, approximately 100 mg of lyophilized venom was dissolved in distilled water to a final concentration of 50 mg/ml (2 ml total volume) and clarified by centrifugation. The supernatant was fractionated by RP-HPLC using a Waters 2795 system (Waters Corporation). Separation was achieved on a semipreparative C18 column (10 mm × 250 mm, 5 μm; Welch Materials Inc.) with a linear acetonitrile gradient from 10% to 55% over 45 min at a flow rate of 3 ml/min. For each purification run, 5 mg of venom was injected. Eluted fractions were collected manually and lyophilized.

The molecular masses of all fractions were determined by MALDI-TOF MS using an AB SCIEX TOF/TOF 5800 instrument (Applied Biosystems). α-Cyano-4-hydroxycinnamic acid was used as the matrix. Spectra were acquired in reflector mode with the following parameters: mass range, 1 to 8 kDa; pulse width, 20 ms; acceleration voltage, 25 kV; and vacuum pressure of 4 × 10^−7^ torr. Mass data from all fractions were compiled for subsequent analysis.

### Venom gland RNA extraction and cDNA library construction

Venom glands were dissected from 10 adult *P. pulcher* spiders with an equal sex ratio, pooled, and immediately snap-frozen in liquid nitrogen. Total mRNA extraction, cDNA synthesis, library construction, and cloning into the pUC19 vector were performed by Shanghai OE Biotech Co., Ltd using the TruSeq Stranded mRNA LT Sample Prep Kit (Illumina, San Diego, CA, USA) and the SMART cDNA Library Construction Kit (Takara Bio). Only cDNA fragments of 400 to 1000 bp were gel-purified and used for library construction. Recombinant clones were plated on Luria–Bertani agar and incubated at 37 °C for 12 h. Individual colonies were randomly selected and sequenced using the universal M13F primer.

### Bioinformatics and sequence analysis

The overall bioinformatics workflow largely followed procedures reported in our previous study (43), with minor modifications as described below. EST data were curated to remove vector- and adapter-derived sequences, and low-quality reads with weak signal or ambiguous base calls were excluded. High-quality ESTs containing a poly(A) tail of ≥10 adenine residues were retained for downstream analysis.

To identify putative toxin-related transcripts, ESTs were queried against the NR protein database using BLASTx (E-value < 1 × 10^−5^). Based on BLAST results, ESTs were classified as toxin-like, cellular component-related, or unannotated. Clustering of each ESTs group was conducted using the SeqMan Pro 11.1.0 module within DNASTAR Lasergene 11.0.7 software suite (DNASTAR, Inc., Madison, WI, USA) (44). Redundant ESTs were assembled into contigs to reconstruct unique precursor sequences. ORFs were predicted using the Expasy translation tool, and the longest ORF in the 5′→3′ orientation containing a stop codon was selected for each precursor.

Sequence relationships among toxin precursors were assessed using ClustalX 2.1, with a focus on conserved cysteine frameworks. Phylogenetic analysis was performed using the Neighbor-Joining method in MEGA 7.0.26, and trees were visualized with iTOL (https://itol.embl.de). Amino acid frequency distributions and consensus motifs for each toxin family were generated using WebLogo 3 (https://weblogo.threeplusone.com/create.cgi). Signal peptides and propeptide–mature peptide cleavage sites were predicted using SignalP 6.0 (https://services.healthtech.dtu.dk/services/SignalP-6.0) and the Processing Quadruplet Motif model, respectively (45). Potential C-terminal amidation was inferred from glycine residues compatible with peptidylglycine α-amidating monooxygenase (PAM) (46). Custom Python scripts implemented in PyCharm (v.2025.2.4) were used for batch processing and cross-checking of sequence.

### Three-dimensional structure prediction

The three-dimensional structures of the representative mature peptides and the peptide–ion channel complexes were predicted using AlphaFold 3 (DeepMind), a deep learning–based protein structure prediction framework capable of modeling protein–protein interaction (47, 48). Disulfide bonds were automatically inferred in PyMOL 2.4.1 (Schrödinger) based on cysteine residue identities and the spatial proximity of sulfur atoms. The complex model with the highest confidence was chosen to analyzed the detailed interactions between the peptide and ion channel using ChimeraX (version 1.7.1) (University of California).

### Chemical synthesis and oxidative refolding of Pp1a

Pp1a was chemically synthesized manually using the Fmoc/tert-butyl strategy and HOBt/TBTU/NMM coupling method, as previously described (49). The CTC resin was used to obtain a free carboxyl group at the C terminus. After cleavage from the resin and removal of side-chain protecting groups, the crude reduced peptide was purified by RP-HPLC. Fractions corresponding to the linear peptide were pooled, concentrated, and lyophilized prior to oxidative refolding. For refolding, 15 mg of the lyophilized linear peptide was first dissolved in ddH_2_O and then diluted into a refolding buffer containing 0.1 M Tris–HCl (pH 7.4), 0.1 M NaCl, 5 mM reduced GSH, and 0.5 mM GSSG, to a final peptide concentration of 0.1 mg/ml. The refolding reaction was carried out at 4 °C with gentle stirring for 12 h. The reaction was terminated by the addition of TFA to a final concentration of 0.1%. The correctly refolded peptide was subsequently isolated by RP-HPLC. The molecular masses of both the linear reduced peptide and the refolded product were confirmed by MALDI-TOF MS using a Shimadzu MALDI-8020 (Shimadzu Corporation).

### Cell culture and transfection

Human embryonic kidney 293T (HEK293T, ATCC CRL-3216) cells were maintained in Dulbecco’s Modified Eagle Medium (Invitrogen; Thermo Fisher Scientific) supplemented with 10% fetal bovine serum and 1% penicillin-streptomycin (both from Gibco; Thermo Fisher Scientific) under standard conditions (37 °C, 5% CO_2_, saturated humidity). When cells reached 80 to 90% confluence, transfection was performed using Lipofectamine 2000 (Invitrogen; Thermo Fisher Scientific) according to the manufacturer’s instructions. Briefly, either 2 μg rat ASIC1a or rat ASIC1a/F350A plasmid, together with 0.5 μg pEGFP-N1 plasmids were cotransfected into HEK293T cells. For molecular docking analyses, the human ASIC1a structure was used as the template, based on its high sequence conservation with the rat channel and the availability of high-resolution structural data. 4 to 6 h posttransfection, cells were seeded onto poly-L-lysine-coated coverslips. Patch-clamp recordings were conducted 24 to 36 h later, with EGFP fluorescence used to indicate successfully transfected cells.

### Electrophysiology

Whole-cell patch clamp recordings were performed using an EPC10 amplifier controlled by Patchmaster v2 × 65 software (HEKA Elektronik). Patch pipettes were prepared from glass capillaries using the PC-10 puller (NARISHIGE) and had a resistance of 2 to 3 MΩ when filled with the intracellular solution. The intracellular solution contains (in mM): 140 KCl, 5 NaCl, 2 MgCl_2_, 5 EGTA, 10 Hepes (pH7.4, adjusted with KOH). The bath (extracellular) solution contains (in mM): 140 NaCl, 5 KCl, 2 MgCl_2_, 2 CaCl_2_, 10 D-Glucose. For solutions adjusted to pH 7.4, 10 mM Hepes was added. For acidic bath solutions with pH 6.0, 5 mM MES and 5 mM Hepes were used. The pH of all bath solutions were adjusted to the desired value using NaOH. The osmolality of both intracellular and bath solutions was adjusted to 300 to 320 mOsm, and all solutions were filtered through a 0.22 μm filter prior to use. All reagents were purchased from Sigma-Aldrich. Fast and slow capacitance effects were sequentially compensated using the C_f_ and C_m_ function of the amplifier. Only cells with a series resistance below 10 MΩ after break-in were included, and series resistance was compensated by 80% to minimize voltage errors.

For recording ASIC1a (WT and F350A mutant) currents, cells were voltage-clamped at −60 mV, and currents were evoked by rapid application of acidic solutions. Only cells exhibiting stable responses to at least three consecutive acid stimulations were included for analysis. Between stimulations, cells were maintained under continuous bath solution flow for 1 min. Acidic and bath solutions were delivered using a constant-flow system controlled by a VC-6 six-channel valve controller (Warner Instruments), with the flow rate maintained at 1 ml/min. To evaluate toxin effects, cells were continuously exposed with toxin-containing bath solution for up to 3 min, and channel currents were subsequently evoked by acidic solutions containing the same toxin concentration.

### Data analysis

Data are presented as mean ± SD, with n indicating the number of independent cells. Analyses were performed using Igor Pro 6.10A (WaveMetrics), Microsoft Excel 2019 (Redmond), and GraphPad Prism 9.5.1 (La Jolla). Dose-response curves were fitted using the Hill equation: Y = Bottom + (Top - Bottom)/(1 + 10ˆ((LogIC_50_-X) ∗ HillSlope)), where IC_50_ represents the half-maximal inhibitory concentration.

## Data availability

The data that have been used are confidential.

## Supporting information

This article contains supporting information (16).

## Conflict of interest

The authors declare that they have no conflicts of interest with the contents of this article.
