# Supplementary material for: Molecular and structural dissection of Psalmopoeus pulcher venom reveals cysteine-rich peptides that target acid-sensing ion channel 1a
Source: J Biol Chem. 2026 Jul 21;302(9):113347. doi: 10.1016/j.jbc.2026.113347 (PMC13488068; doi:10.1016/j.jbc.2026.113347)
Supplement: Supplementary file 1 [file mmc1.docx]

**Supporting information**

**Supplementary figure 1. Comparative analysis of toxin precursor characteristics in *Psalmopoeus* spiders (*P. pulcher* and *P. reduncus*).** *A*, Number of toxin precursor identified in *P. pulcher* and *P. reduncus,* classified according to the presence or absence of an N-terminal methionine residue (Met-start and non–Met-start). *B*, Percentage distribution of toxin precursor in amino acid lengths. *C*, Pairwise sequence identity distribution between *P. pulcher* and *P. reduncus* toxin precursor. The *P. reduncus* toxin precursor dataset was compiled from Supplementary Data 3 reported by Lüddecke et al. (16). Only sequences annotated as hexatoxin, neurotoxin, uCNTX, undetermined neurotoxin, hainantoxin, MIT atracotoxin, and psalmotoxin-1 were included in the analysis. Analyses shown in panels B and C were restricted to precursor sequences containing an N-terminal methionine residue.

**Supplementary figure 2. Molecular docking analysis of the predicted mature peptide derived from PpTx-247 with human Kv1.3 and human Kir6.2 using AlphaFold 3**. The predicted mature peptide of PpTx-247 is shown in dark gray, whereas the channel subunits are shown in different colors. Key interacting residues are displayed as side chains and labeled for clarity

**Supplementary figure 3. Characterization of native and synthetic Pp1a by mass spectrometry and co-elution analysis**

*A*, Full MALDI-TOF mass spectrum of the high-purity native Pp1a fraction, corresponding to the inset shown in Fig. 6*A*. *B*, MALDI-TOF mass spectrum of linear reduced Pp1a. *C*, MALDI-TOF mass spectrum of refolded synthetic Pp1a, corresponding to the red-star marked peak in Fig. 6*C*. *D*, Co-elution chromatogram of native and refolded synthetic Pp1a.

**Supplementary Table 1****. Predicted 3D structures of representative mature peptides derived from Family K and selected ion channels by AlphaFold 3.** Known peptide toxins were included as positive controls for each channel. Intracellular denotes binding poses within intracellular channel domains and was considered non-physiological.

| Ion channel | Peptide toxin | iptm | ptm | Ranking score | Comment |
| --- | --- | --- | --- | --- | --- |
| Kv1.3 | Shk | 0.81 | 0.81 | 0.86 | Positive control peptide |
|  | PpTx-47 | 0.75 | 0.75 | 0.80 | intracellular |
|  | PpTx-166 | 0.71 | 0.73 | 0.77 | intracellular |
|  | PpTx-244 | 0.77 | 0.78 | 0.83 | - |
|  | PpTx-247 | 0.74 | 0.75 | 0.75 | - |
| Cav2.2 | Ziconotide | 0.86 | 0.59 | 0.8 | Positive control peptide |
|  | PpTx-47 | 0.27 | 0.65 | 0.45 | intracellular |
|  | PpTx-166 | 0.26 | 0.65 | 0.45 | intracellular |
|  | PpTx-244 | 0.23 | 0.65 | 0.42 | intracellular |
|  | PpTx-247 | 0.27 | 0.65 | 0.45 | intracellular |
| Nav1.2 | KIIIA | 0.96 | 0.73 | 1.06 | Positive control peptide |
|  | PpTx-47 | 0.36 | 0.70 | 0.56 | - |
|  | PpTx-166 | 0.23 | 0.69 | 0.46 | - |
|  | PpTx-244 | 0.27 | 0.70 | 0.49 | - |
|  | PpTx-247 | 0.25 | 0.69 | 0.48 | - |
| Kir1.1 | SsTx-4 | 0.74 | 0.76 | 0.82 | Positive control peptide |
|  | PpTx-47 | 0.71 | 0.73 | 0.79 | - |
|  | PpTx-166 | 0.69 | 0.71 | 0.77 | - |
|  | PpTx-244 | 0.69 | 0.71 | 0.77 | - |
|  | PpTx-247 | 0.70 | 0.72 | 0.78 | - |
| Kir4.1 | SsTx-4 | 0.77 | 0.78 | 0.83 | Positive control peptide |
|  | PpTx-47 | 0.69 | 0.70 | 0.77 | - |
|  | PpTx-166 | 0.66 | 0.68 | 0.75 | - |
|  | PpTx-244 | 0.67 | 0.69 | 0.75 | - |
|  | PpTx-247 | 0.66 | 0.68 | 0.75 | - |
| Kir6.2 | SsTx-4 | 0.74 | 0.75 | 0.85 | Positive control peptide |
|  | PpTx-47 | 0.68 | 0.70 | 0.77 | - |
|  | PpTx-166 | 0.67 | 0.69 | 0.75 | - |
|  | PpTx-244 | 0.74 | 0.75 | 0.84 | - |
|  | PpTx-247 | 0.74 | 0.76 | 0.84 | - |

**Supplementary figure 1**

**Supplementary figure 2**

**Supplementary figure 3**
